# Targeting of Prosurvival Pathways as Therapeutic Approaches against Primary Effusion Lymphomas: Past, Present, and Future

**DOI:** 10.1155/2015/104912

**Published:** 2015-01-28

**Authors:** Marisa Granato, Roberta Santarelli, Roberta Gonnella, Antonella Farina, Pankaj Trivedi, Alberto Faggioni, Mara Cirone

**Affiliations:** Department of Experimental Medicine, Istituto Pasteur Fondazione Cenci Bolognetti, Sapienza University, 00161 Rome, Italy

## Abstract

Constitutively activated prosurvival pathways render cancer cells addicted to their effects. Consequently they turn out to be the Achilles' heels whose inhibition can be exploited in anticancer therapy. Primary effusion lymphomas (PELs) are very aggressive non-Hodgkin's B cell lymphomas, whose pathogenesis is strictly linked to Kaposi's sarcoma herpesvirus (KSHV) infection. Here we summarized previous studies from our and other laboratories exploring the cytotoxic effect of drugs inhibiting the main prosurvival pathways activated in PEL cells. Moreover, the immunogenicity of cell death, in terms of dendritic cell (DC) activation and their potential side effect on DCs, is discussed.

## 1. Primary Effusion Lymphomas' Biology

Primary effusion lymphomas (PELs) are very aggressive non-Hodgkin's B cell lymphomas with poor prognosis that frequently arise in immune-compromised patients [1]. PELs originate from postgerminal center B cells and exhibit indeterminate immune-phenotypes; that is, they express CD45, CD138, and activation-associated antigens (CD30, CD38, and HLA-DR) and lack surface expression of B cell markers (CD19, CD20, CD79a, and immunoglobulin) but exhibit clonal rearrangements and somatic hypermutation of the immunoglobulin genes. PELs are strictly associated with a DNA oncovirus, the Kaposi's sarcoma herpesvirus (KSHV), and, in the majority of cases, they are dually infected by a second oncovirus belonging to the gamma-herpesvirus family [2, 3], the Epstein-Barr virus (EBV) [4]. The low response to conventional therapies indicates that new therapeutic strategies are needed for PELs. PEL tumor growth relays on the presence of cytokines that, produced by the tumor itself [5], stimulate their own release with an autocrine positive feedback loop. An important role in PEL survival has been reported for interleukin- (IL-) 6, IL-10, vascular endothelial growth factor (VEGF), and Oncostatin M [6–8]. In addition to cellular cytokines, KSHV encodes its own viral cytokines such as IL-6 which is similar to human IL-6 and exerts an important prosurvival effect on PEL cells [6, 9] and macrophage inhibitory proteins (MIPs) that affect the immune system of the infected host [10].

## 2. Prosurvival Molecules in PELs

PEL cell survival relays on the constitutive activation of several pathways and also on the hyperexpression of several heat shock proteins (HSPs), a characteristic that is common among the cancer cells.

### 2.1. JAK2/STAT3 Pathway

By engagement of their specific cellular receptors, cytokines such as IL-6 and IL-10 and VEGF activate the JAK2/STAT3 transduction pathway that promotes the transcription of genes encoding for proteins involved in PEL resistance to apoptosis and contributes to tumor survival [11–13]. Consequently, the specific inhibition of STAT3 activation by AG490 has been reported to induce PEL cell death by downregulating survivin expression [14]. In addition, we have recently reported that AG490 is able to trigger an immunogenic apoptosis in PEL cells [15]. This aspect is of pivotal importance since it is now clear enough that, without the contribution of the immune system, obtained by triggering an immunogenic cell death type and/or the reversion of the tumor induced immunosuppression [16], no complete eradication of cancer can be achieved by anticancer therapies [17, 18]. Moreover AG490 is able to revert immunosuppression caused by tumor-released factors or induced by tumor viruses such as KSHV, which correlates with STAT3 activation in immune cells [19, 20]. This immune-stimulating AG490 property further encourages its use in PEL anticancer therapy.

### 2.2. PI3K/AKT/mTOR Pathway

It is known that, in addition to STAT3, cytokines like IL-6 and IL-10, VEGF, and Oncostatin M [21] are able to activate other pathways such as PI3K/AKT and Ras/MAPK that govern fundamental processes [22], such as cell proliferation, differentiation, metabolism, and tumor survival. The PI3K/AKT is constitutively activated in PELs due to the effect mediated by PEL released cytokines and following KSHV infection [23], in particular as an effect of K1 viral protein expression [24]. Previous papers have reported that targeting PI3K/AKT pathway in PEL cells may represent an effective anticancer strategy [25, 26]. We have reported that KSHV infection hyperactivates AKT in THP-1 cells and, as a consequence, the response to proteasome inhibition is reduced. Moreover, AKT activation also leads to Glut1 membrane localization, which is known to promote cell survival by increasing the glucose uptake. On the other hand, we found that Glut1 translocation renders THP-1 cells more susceptible to the glycolysis inhibitor 2-deoxy-D-glucose (DG) effect [27]. This is another example of how a prosurvival effect turns out to be a prodeath effect in tumor cells. Finally, since PI3K/AKT activation leads to the phosphorylation of the downstream molecule mTOR, also its inhibition has been exploited in PEL therapy [28]. mTOR inhibition has been shown to reduce PEL cell survival by interfering with the release of cytokines known to be essential for PEL cell growth, and more recently it has been shown that mTOR inhibition can be effective also against a second KSHV-associated malignancy such as Kaposi's sarcoma.

### 2.3. MAPK Pathways

Mitogen activated protein kinases (MAPKs) including ERKs, JNKs, and p38 kinases control a vast array of physiological processes [29]. They can be activated in tumor cells by several stimuli such as stress conditions and inflammatory cytokines, also produced by tumor itself. Their inhibition can be potentially explorable in PEL therapy, considering that a cross-talk between these pathways and other prosurvival pathways, such as NF-*κ*B and PI3K/AKT/m-TOR, has been reported [30]. In a recent study, we have shown that JNK inhibition enhances Bortezomib-induced cell death and that SP600125 JNK inhibitor is also able to induce PEL cell death to some extent [31]. A prosurvival role of JNK2 activation during the ER stress caused by tunicamycin treatment has also been shown in a different cell type [32]. In addition, p38 MAPK seems to be activated in PEL cells, in particular in those harbouring KSHV, in comparison with KSHV-negative PEL cells [4].

### 2.4. NF-*κ*B Pathway

An essential role in PEL cell survival is known to be played by NF-*κ*B, constitutively activated in PEL cells due also to the effect mediated by KSHV-encoded viral FLICE-inhibitory protein (v-FLIP) expression [33, 34]. It has been reported that NF-*κ*B inhibition with Bay11-7082 exerts a strong reduction of PEL cell survival [33]. Moreover, the Bortezomib cytotoxic effect, previously observed on PEL cells, also involves NF-*κ*B inhibition [35], even if its main cytotoxic effect is proteasome inhibition [36]. We have previously explored the immunogenicity of Bortezomib-induced cell death in PEL and showed that Bortezomib was able to induce damage associated molecular patterns (DAMPs) expression on the surface of apoptotic PEL cells, which then resulted in dendritic cell (DC) activation [15, 37]. Moreover, we have demonstrated that Bortezomib induces endoplasmic reticulum (ER) stress and a prosurvival autophagy in PEL cells, due to the accumulation of ubiquitinated proteins consequent to the inhibition of proteasomal degradation. The autophagic blockage, during Bortezomib treatment, further increased its cytotoxic effect. Thus this strategy could be explored in the cancer therapy against PELs [31].

### 2.5. HSPs

A further effect mediated by the activation of pathways such as Ras/MAPK and JAK/STAT3 is the increase of heat shock protein (HSP) expression [38, 39]. HSPs are known to help cancer cells to survive in the stressful conditions caused by their rapid growth and nutrient shortage. HSPs also counteract the cytotoxic effects induced by chemotherapeutic treatments. For this reason they represent a target for anticancer therapy [38], also considering that their expression, and consequently the cytotoxic effect of their inhibition, is low in normal cells compared with cancer cells. Accordingly, we have recently reported that the inhibition of HSP70, using the small molecule 2-phenylethynesulfonamide (PES), is a successful therapeutic strategy against PEL cells and that PES showed very low cytotoxic effect on normal B cells from which PEL cells arise [40]. PES induced lysosome permeabilization and a necroptotic cell death type in PEL cells with immunogenic properties toward DCs. It represents a valid strategy against this cancer and possibly against other tumors that, displaying oncosuppressor mutations, may be resistant to apoptosis inducing drugs. Also the inhibition of HSP90, another chaperone protein with the essential function in protein correct folding, has been reported to be effective against PELs [41, 42], as well as against multiple myeloma, which shares many similarities with PELs [43, 44].

## 3. Antiviral Strategies in PELs

PEL cells harbour KSHV, which persists in a latent state in the majority of the cells. Upon appropriate stimuli, latent infection can be switched into lytic productive infection that generally leads to cell lysis and spread of the viral particles. The possibility to kill tumor cells by inducing viral replication can be explored in the therapy against tumors latently infected with DNA viruses, especially because during viral replication tumor cells become sensitive to the effects induced by the antiviral drugs. For the above reasons, strategies that allow the manipulation of viral life cycle, reducing or promoting viral production, are potentially explorable in PELs. One of the strategies that allow manipulating the viral life cycle is the modulation of the cellular autophagy which has been shown to have a strong impact on KSHV replication [45].

## 4. Experimental Data

**Figure 1:**
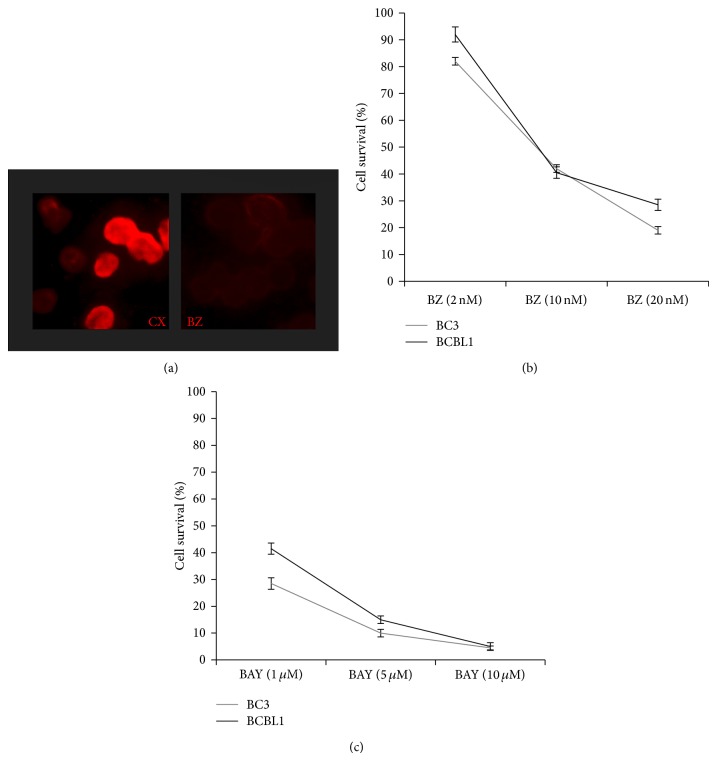
Effect of Bortezomib (BZ) (Santa Cruz) or Bay11-7082 (BAY) (Santa Cruz) on BC3 and BCBL1 PEL cells. (a) Effect on NF-*κ*B p65 nuclear localization of Bortezomib in BC3 cells, in comparison to the untreated control cells, analyzed by immunofluorescence assay (IFA). A representative experiment out of three is shown. (b and c) Trypan blue exclusion assays of BC3 and BCBL1 PEL cells treated for 24 hours with Bortezomib (BZ) or Bay11-7082 (BAY), at the indicated doses. The mean ± SD of three independent experiments is reported.

**Figure 2:**
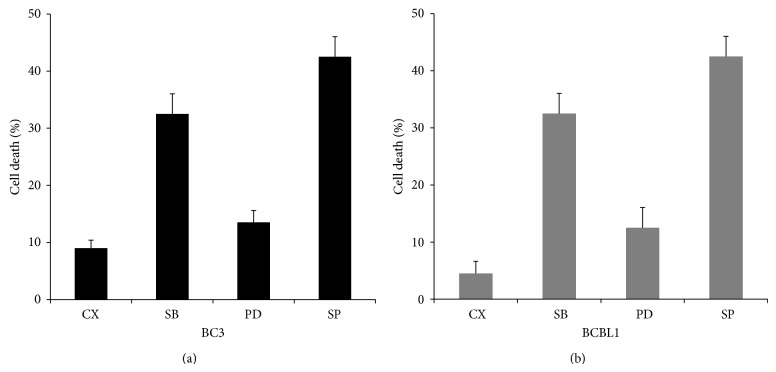
Effect on PEL cell cytotoxicity of p38, JNK, or ERK inhibition by SB203580, SP600125, and PD98059, respectively, based on trypan blue exclusion is reported. The treatment was performed for 24 hours at the concentration of 10 *μ*M. All the reagents were purchased from Santa Cruz. The mean ± SD of three independent experiments is reported.

Here we have summarized our and other experimental evidences showing the cytotoxic effect mediated by the pharmacological inhibition of all these pathways in PEL cell lines. We first confirmed that indirectly inhibiting NF-*κ*B with Bortezomib (Figure 1(a)) a dose-dependent reduction of PEL cell survival can be observed (Figure 1(b)), confirming our previous reported results [15]. Even stronger cytotoxic activity on PEL cells was obtained with Bay11-7082 NF-*κ*B, specific inhibitor [46], that was indeed able to kill the majority of PEL cells at the concentration of 5 mM (Figure 1(c)). Next, given the importance of JAK2/STAT3 activation in PEL survival and in the reversion of immunosuppression, reported by us as well as by other groups [14, 15, 47], we performed a dose-response assay pharmacologically inhibiting STAT3 activation with AG490 and confirmed that it was very effective in reducing PEL cell survival. Besides its efficacy against PEL cells, the use of AG490 is also encouraged by our previous observations showing that it can induce an immunogenic cell death in these cells and has a low side effect on dendritic cell viability [15, 38]. Next, a dose-response treatment aimed at the targeting of PI3K/AKT pathway was then performed with AKT inhibitor LY294002 against BC3 and BCBL1 PEL cells. LY294002, used at the concentration of 20 mM, induced about 50% reduction of PEL cell survival, after 24 hours of treatment (Figure 2). One of the consequences of AKT activation is a change in the cell metabolism, such as an increase of cell resistance to glycolysis inhibitors. Thus, it will be interesting to investigate how treatment LY294002 would affect PEL cell resistance to glucose starvation. A low cytotoxic effect on PEL cells was observed with rapamycin mTOR inhibitor, used at 50 nM, for 24 hours. However, its combination with autophagy inhibitor 3-methyladenine (3-MA) resulted in a higher cytotoxic effect (unpublished data). This is in agreement with the notion that inhibition of mTOR induces autophagy that usually helps cells to survive during starvation or stressful conditions; thus its inhibition may increase cell death.

**Figure 3:**
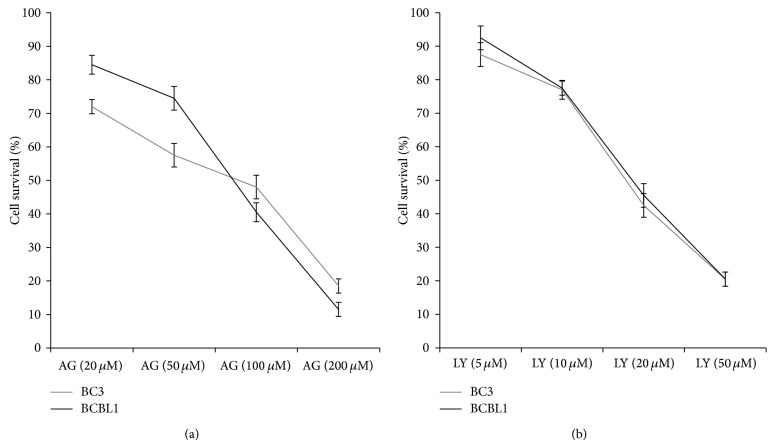
Dose-response cytotoxic assay of STAT3 or AKT inhibition in BC3 and BCBL1 PEL cells treated with AG490 (AG) (Calbiochem) or LY294002 (LY) (Santa Cruz), respectively, at the indicated doses. Trypan blue exclusion assays were performed after 24 hours of treatment. Mean ± SD of three experiments is reported.

Additionally, the potential cytotoxic effect of the inhibition of JNK, p38, and ERK MAPK pathways was evaluated on PEL cells. The results shown in Figure 3 indicate that the higher cytotoxic effect was obtained by using SP600125 JNK inhibitor, confirming that this pathway plays an important prosurvival role in PEL cells, according to previously reported studies [31]. All MAPK inhibitors were used at the concentration of 10 mM and a low cytotoxicity was also observed with the inhibitor of p38 MAPK SB203580 and with the ERK inhibitor PD98059, according to a recent study [48] (Figure 3).

**Figure 4:**
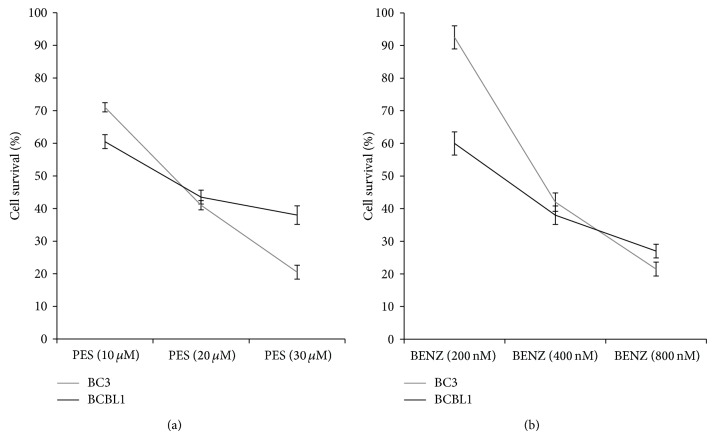
Dose-response cytotoxic assay of BC3 and BCBL1 PEL cells treated with PES (HSP70 inhibitor) (Calbiochem) and Benzisoxazole (BENZ) (HSP90 inhibitor) (Calbiochem), at the indicated doses. Trypan blue exclusion assays were performed after 24 hours of treatment and mean ± SD of three independent experiments is reported.

The activation of the above-mentioned prosurvival pathway leads to upregulation of HSPs, which are classified based on their molecular weight. They play multiple roles in cancer cells; for example, they ensure the correct protein folding, which is very important especially for cancer cells. Moreover, HSPs are required for the expression of some KSHV essential proteins [49, 50]. The major role in cancer cell survival is played by HSP70 and HSP90. The cytotoxicity of HSP70 inhibitor 2-phenylethynesulfonamide (PES) against PEL cells was previously reported by our group [40]. Here we compared the effect of PES with Benzisoxazole HSP90 inhibitor, considering that HSP70, besides its chaperone function, plays an important role in the maintenance of the lysosome membrane stabilization. The results shown in Figure 4 indicate that both HSP70 and HSP90 were essential for PEL cell survival. Trypan blue exclusion was used in all the dose-response cytotoxic assay performed in this study.

**Figure 5:**
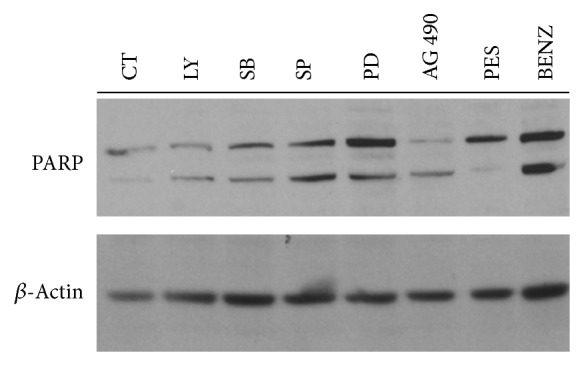
Western-blot analysis showing the PARP cleavage in BCBL1 cells treated for 24 hours with all the above reported cytotoxic drugs: LY294002 (LY), SB203580 (SB), SP600125 (SP), PD98059 (PD), AG490 (AG), PES, and Benzisoxazole (BENZ). *β*-Actin is included as loading control and a representative experiment out of three is reported. Antibodies against PARP (Cell Signaling) and *β*-actin (Sigma Aldrich) were diluted 1 : 1000 and 1 : 10000, respectively.

To further explore how cell death occurred in PEL cells by the inhibition of the above-reported prosurvival pathways, we then performed a western-blot analysis of the poly (ADP-ribose) polymerase (PARP) cleavage. PARP cleavage generally represents a final event of an apoptotic cell death and is mainly mediated by caspase activation [51]. We found that, except for PES, previously shown to induce a necroptotic cell death type [40], all the other drugs utilized in this study induced the cleavage of PARP in PEL cells (Figure 5). Besides considering the cytotoxic effect, it is important to evaluate the side effect that anticancer drugs could have on the immune cells and DCs in particular, being cells with a pivotal role in the immune system [52]. Preliminary data obtained by exposing monocyte-derived DCs to all the drugs used against PEL cells, at the concentrations able to reduce 50% of PEL cell survival, showed that all of them, except for Bortezomib and Bay11-7082, were almost completely safe towards DCs (data not shown).

## 5. Conclusion

In conclusion, the use of drugs that target constitutively activated pathways in PEL cells could represent a valid alternative in translational therapies, especially considering the ability of most of them to induce an immunogenic cell death. In addition, their use is very promising because they have a low side effect towards the immune cells, and DCs in particular, rendering their use safer than conventional chemotherapies.
